# Machine learning unveils an immune-related DNA methylation profile in germline DNA from breast cancer patients

**DOI:** 10.1186/s13148-024-01674-2

**Published:** 2024-05-15

**Authors:** Ning Yuan Lee, Melissa Hum, Guek Peng Tan, Ai Choo Seah, Pei-Yi Ong, Patricia T. Kin, Chia Wei Lim, Jens Samol, Ngiap Chuan Tan, Hai-Yang Law, Min-Han Tan, Soo-Chin Lee, Peter Ang, Ann S. G. Lee

**Affiliations:** 1 Division of Cellular and Molecular Research, National Cancer Centre Singapore, 30 Hospital Boulevard, Singapore, 168583 Republic of Singapore; 2 DNA Diagnostic and Research Laboratory, KK Women’s and Children’s Hospital, 100 Bukit Timah Rd, Singapore, 229899 Singapore; 3 SingHealth Polyclinics, 167 Jalan Bukit Merah Connection One (Tower 5), Singapore, 150167 Singapore; 4 Department of Hematology-Oncology, National University Cancer Institute, Singapore (NCIS), National University Health System, 5 Lower Kent Ridge Road, Singapore, 119074 Singapore; 5 Department of Personalised Medicine, Tan Tock Seng Hospital, 11 Jalan Tan Tock Seng, Singapore, 308433 Singapore; 6 Medical Oncology Department, Tan Tock Seng Hospital, 11 Jalan Tan Tock Seng, Singapore, 308433 Singapore; 7 Johns Hopkins University, Baltimore, MD 21218 USA; 8 SingHealth Duke-NUS Family Medicine Academic Clinical Programme, Duke-NUS Medical School, 8 College Road, Singapore, 169857 Singapore; 9 Lucence Diagnostics Pte Ltd, 211 Henderson Road, Singapore, 159552 Singapore; 10 Department of Medicine, Yong Loo Lin School of Medicine, National University of Singapore, 10 Medical Dr, Singapore, 117597 Singapore; 11 Cancer Science Institute, Singapore (CSI), National University of Singapore, 14 Medical Dr, Singapore, 117599 Singapore; 12 Oncocare Cancer Centre, Gleneagles Medical Centre, 6 Napier Road, Singapore, 258499 Singapore; 13 SingHealth Duke-NUS Oncology Academic Clinical Programme (ONCO ACP), Duke-NUS Graduate Medical School, 8 College Road, Singapore, 169857 Singapore; 14 Department of Physiology, Yong Loo Lin School of Medicine, National University of Singapore, 2 Medical Drive, Singapore, 117593 Singapore

**Keywords:** Breast cancer, DNA methylation, Peripheral blood, Early detection, Liquid biopsy, Biomarker, Machine learning

## Abstract

**Background:**

There is an unmet need for precise biomarkers for early non-invasive breast cancer detection. Here, we aimed to identify blood-based DNA methylation biomarkers that are associated with breast cancer.

**Methods:**

DNA methylation profiling was performed for 524 Asian Chinese individuals, comprising 256 breast cancer patients and 268 age-matched healthy controls, using the Infinium MethylationEPIC array. Feature selection was applied to 649,688 CpG sites in the training set. Predictive models were built by training three machine learning models, with performance evaluated on an independent test set. Enrichment analysis to identify transcription factors binding to regions associated with the selected CpG sites and pathway analysis for genes located nearby were conducted.

**Results:**

A methylation profile comprising 51 CpGs was identified that effectively distinguishes breast cancer patients from healthy controls achieving an AUC of 0.823 on an independent test set. Notably, it outperformed all four previously reported breast cancer-associated methylation profiles. Enrichment analysis revealed enrichment of genomic loci associated with the binding of immune modulating AP-1 transcription factors, while pathway analysis of nearby genes showed an overrepresentation of immune-related pathways.

**Conclusion:**

This study has identified a breast cancer-associated methylation profile that is immune-related to potential for early cancer detection.

**Supplementary Information:**

The online version contains supplementary material available at 10.1186/s13148-024-01674-2.

## Introduction

The earlier breast cancer is detected, the better the treatment outcome [1]. Current technologies of early detection such as screening mammograms or clinical breast examinations still suffer from costly false positives and overdiagnoses [2–4]. Blood-based biomarkers of cancer show great promise in supplementing or even replacing these technologies for early detection: there are already commercially available blood-based diagnosis kits for various cancers, including breast cancer [5]. Here, we focus on peripheral blood DNA methylation, as it is easy to collect and process especially relative to cell-free DNA.

However, the search for an accurate peripheral blood DNA methylation profile for breast cancer is still far from complete [6]. Hitherto many studies on breast cancer have examined the 450,000 CpGs profiled by the HumanMethylation450 array in large-scale nested case–control studies [7–10], but to the best of our knowledge there have not been any large-scale studies searching the 850,000 CpGs profiled by the MethylationEPIC array. Furthermore, these studies investigate predominantly European populations. Since methylation is influenced by environment [11–13] and heritable to a certain extent [14, 15], it is not clear whether these previously identified breast cancer-associated methylation profiles are applicable also in other populations of different ancestries in different environments. Finally, while the mechanism behind the association between cell-free DNA and various cancers is likely via circulating tumor DNA [16], it is less clear which mechanisms drive the association of certain whole blood methylation profiles—comprising mostly of DNA from circulating blood cells—with breast cancer.

Herein we present the largest-to-date epigenome-wide study of breast cancer-associated methylation profiling in an Asian population, to the best of our knowledge. By profiling 850,000 CpGs for each breast cancer patient or healthy control, we identify a peripheral blood DNA methylation profile which can distinguish breast cancer patients from healthy controls when used in various machine learning algorithms. We benchmark this methylation profile alongside four breast cancer-associated methylation profiles previously identified in predominantly European populations. Enrichment analyses suggest a link between activated immune cells and this newly identified breast cancer peripheral blood DNA methylation profile.

## Methods

### Study participants

**Fig. 1:**
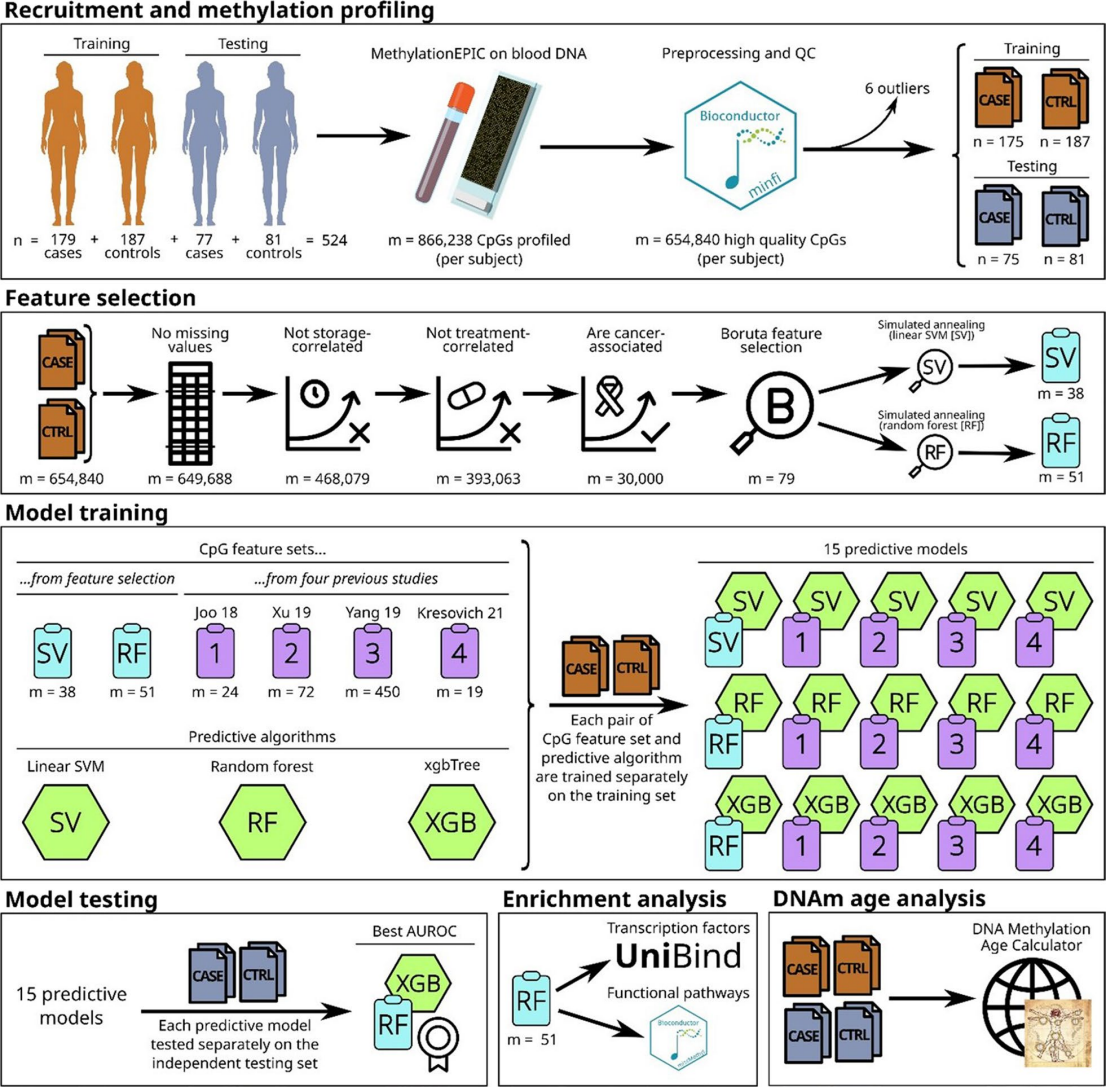
Overview of this study. The number of CpGs at each step is indicated by “m = …”

This study included a total of 524 female subjects of Chinese ethnicity, consisting of 256 breast cancer patients (affected) and 268 non-cancer controls (unaffected). The clinicopathological characteristics of the patients are shown in Table 1. Peripheral blood samples from breast cancer patients were collected from multiple sites in Singapore, namely the National Cancer Centre Singapore (NCCS), National University Hospital (NUH), Tan Tock Seng Hospital (TTSH), and Lucence Diagnostics. The unaffected controls were recruited from the SingHealth Outram and Bukit Merah Polyclinics (*n* = 130) and KK Women’s and Children’s Hospital in Singapore (*n* = 138). Peripheral blood samples were obtained from participants undergoing routine mammogram screening at SingHealth Polyclinics, all of whom were negative for breast cancer. DNA samples from KK Women’s and Children’s Hospital were archival samples acquired from the DNA Diagnostic and Research Laboratory, which originated from the National Thalassemia Registry where blood samples were collected to screen for Thalassemia. Inclusion criteria for the breast cancer patients were that they were Chinese, eligible for genetic testing, and were *BRCA*-negative. Unaffected controls were selected from healthy females with no prior history of cancer and were individually matched with affected cases based on age (± 5 years). The study cohorts were divided into a training cohort, comprising 179 affected patients and 187 unaffected controls, and a testing cohort of 77 affected cases and 81 unaffected controls. An overview of our study design is shown in Fig. 1. Written informed consent was obtained from all participants, and this study was approved by the SingHealth Centralized Institutional Review Board (CIRB Ref: 2018/2147 and 2018/2874).

**Table 1:** Clinical characteristics of breast cancer patients and non-cancer controls

Characteristics	Training set		Test set	
	Cancer (*n* = 175)	Control (*n* = 187)	Cancer (*n* = 75)	Control (*n* = 81)
*Age at breast cancer diagnosis*				
Median age, years (range)	39 (22–72)	40 (22–72)	39 (19–69)	40 (19–69)
≤ 40	111 (63%)	98 (52%)	48 (64%)	43 (53%)
≥ 41	64 (37%)	89 (48%)	27 (36%)	38 (47%)
*Personal history of breast cancer*				
Unilateral	164 (94%)	n/a	73 (97%)	n/a
Bilateral	11 (6%)	n/a	2 (3%)	n/a
*Histology*				
Ductal carcinoma in situ (DCIS)	13 (7%)	n/a	4 (5%)	n/a
Infiltrating ductal carcinoma (IDC)	109 (63%)	n/a	44 (59%)	n/a
Infiltrating lobular carcinoma (ILC)	6 (3%)	n/a	3 (4%)	n/a
Mucinous carcinoma	4 (2%)	n/a	1 (1%)	n/a
Medullary carcinoma	1 (1%)	n/a	1 (1%)	n/a
Invasive micropapillary carcinoma (IMC)	2 (1%)	n/a	0	n/a
Invasive carcinoma (NST)	7(4%)	n/a	3 (4%)	n/a
Others	7 (4%)	n/a	5 (7%)	n/a
Subtype not defined^a^	26 (15%)	n/a	14 (19%)	n/a
*Family history of any cancers (n* = *172)*				
At least first-degree	82 (47%)	n/a	23 (31%)	n/a
At least second-degree	33 (19%)	n/a	21 (28%)	n/a
Third-degree	5 (3%)	n/a	4 (5%)	n/a
Unspecified	3 (2%)	n/a	1 (1%)	n/a
*Family history of breast cancer*^*b*^* (n* = *110)*				
At least first-degree	55 (31%)	n/a	15 (20%)	n/a
At least second-degree	18 (10%)	n/a	11 (15%)	n/a
Third-degree	4 (2%)	n/a	4 (5%)	n/a
Unspecified	3 (2%)	n/a	0	n/a
*Recorded treatment history (n* = *179)*				
Had surgery	43 (25%)	n/a	18 (24%)	n/a
Had radiotherapy	20 (11%)	n/a	9 (12%)	n/a
Had chemotherapy	34 (19%)	n/a	15 (20%)	n/a
Had hormone therapy	29 (17%)	n/a	11 (15%)	n/a
*Blood storage duration*				
Median, days	2057	1063	1722	1088
Min–Max, days	292–7327	457–2589	322–7041	457–1582

^a^Clinical information for some patients was unavailable from one of the sites of this study due to the Institutional Review Board (IRB) approval obtained

^b^In some cases, patients have a family history of breast cancer as well as other types of cancer

### DNA extraction

Genomic DNA was extracted from whole blood or buffy coat using the QIAamp DNA Blood Kit (Qiagen, Hilden, Germany) according to the manufacturer’s instructions. DNA concentration was determined using QuantiFluor dsDNA system (Promega, Madison, WI), and fluorescence readings at 504nmEx/531nmEm were measured using a 96-well plate reader (TECAN, Austin). DNA quality was assessed using a Nanodrop ND-1000 spectrophotometer (Thermo Scientific).

### Epigenomic profiling

The DNA methylation profiles of peripheral blood samples were analyzed using the Infinium MethylationEPIC array (Illumina, San Diego, CA), which provides comprehensive coverage of over 850,000 CpG sites. A minimum of 600 ng of genomic DNA obtained from each participant was sent to Macrogen, Inc (Korea) for the EPIC microarray analysis. Genomic DNA was subjected to bisulfite conversion using the EZ DNA methylation kit (Zymo Research, Irvine, CA). The resulting bisulfite-converted DNA was amplified, hybridized onto MethylationEPIC bead chips, and scanned using the Illumina iScan scanner, following standard Illumina procedures.

### Preprocessing

Microarray data were processed using the minfi *R/Bioconductor* package [17]. Probes located near known SNPs of any frequency, or probes known to be cross-reactive were removed. Then, each sample was normalized independently via intra-sample BMIQ normalization [18].

Patient-samples that were outliers in the principal component analysis (PCA) plot (Additional file 1: Figure S1) or in predicted cell-type composition were removed (Additional file 1: Figure S2, estimated by the minfi *estimateCellCounts* function implementing a regression calibration approach on the subset of EPIC probes shared with the Illumina 450k microarray with default settings [19]). Outliers in PCA were defined as those being three interquartile ranges (IQRs) lower than the first quartile, or three IQRs above the third quartile; for both PCA1 and PCA2 axes. Outliers in cell-type composition were identified as those with extreme cell-type compositions: for example, the complete absence of granulocytes.

Patient-samples were partitioned into training and testing sets in a 70–30 split such that the training and testing sets were matched in both their mean ages and in the proportion of affected patient-samples in the training or testing set with treatment data. After the removal of outliers, there were 175 affected cases and 187 unaffected controls in the training set, and 75 affected cases and 81 unaffected controls in the testing set.

### Feature selection

Feature selection was performed strictly on patient-samples of the training set only, using their BMIQ-normalized methylation M-values.

First, CpG sites which were correlated with duration in storage of the DNA sample, or with any treatment were removed. Correlation with the duration in storage was tested using a regression model as implemented by the limma *R/Bioconductor* package [20], where methylation *M*-values was the outcome, and the log-transformed duration was the predictor. Any CpG site with unadjusted *p*-value < 0.05 was removed. Likewise, treatment correlation was tested in the same manner, but with log-transformed time since last treatment as the predictor, and each test was repeated for each of four treatment types: surgery, chemotherapy, hormone, and radiotherapy. Any CpG site with unadjusted *p*-value < 0.05 in any of the four tests was removed.

Next, CpG sites that were correlated with the affected/unaffected condition of the patient-sample were selected. Condition-correlation was tested using a limma regression model where methylation M-values was the outcome, and condition was the predictor. The top 30,000 CpG sites, ranked by unadjusted *p*-values, were selected.

Finally, CpG sites undergo boruta feature selection followed by simulated annealing with random forest or linear support vector machine (SVM) to yield the final list of features [21]. Simulated annealing was performed using the caret *R* package [22], optimizing for the area under the receiver operating characteristic curve (ROC AUC) in a random forest, using tenfold cross-validation, setting the maximum number of iterations without improvement as 20, initial proportion of features as 0.8, perturbation *p* as 0.75, for 512 iterations; all other parameters were left at their default values. We did not perform simulated annealing with xgbTree as each xgbTree iteration took much longer than a random forest or linear SVM iteration such that the overall time required for simulated annealing with xgbTree was unfeasibly large.

To benchmark our methylation profiles, four previous articles on breast cancer-associated blood methylation were identified from the literature [7–10]. Methylation values for CpGs of methylation profiles from these previous studies were extracted from the BMIQ-normalized methylation M-values of our cohort without any CpG-removing preprocessing steps, in order to maximize the number of available CpGs for analysis. Nonetheless, though most CpGs from those previous studies were also measured in our study, some are missing due to differences in the HumanMethylation450 BeadChip used by all four previous studies, and the MethylationEPIC BeadChip used in our study (Additional file 2: Table S1, Additional file 1: Figure S3). Three of the four methylation profiles comprise only CpGs; though one included five “DNA methylation (DNAm) estimators,” each representing a single numeric value computed from many CpGs, quantifying a phenotype such as age acceleration or abundance of monocytes.

### DNA methylation estimators

The five DNAm estimators (PhenoAgeAccel, RajAgeAccel, CD8T, Mono, and CD8pCD28nCD45RAn) used in one of the methylation profiles from previous studies were obtained from the DNA Methylation Age Calculator [23], accessed 24 May 2023. The DNA methylation age (DNAmAge) from the DNA Methylation Age Calculator was also used to compare the DNA methylation age acceleration of affected cases and unaffected controls.

### Model training and evaluation

Using caret, the random forest-simulated annealing methylation profile and methylation profiles from previous studies were each used to train a random forest with optimal mtry values, or xgbTree with maximum depth of six and 1,000 rounds; optimizing for ROC AUC in tenfold cross-validation using the training set only. In addition, the linear SVM-simulated annealing methylation profile and methylation profiles from previous studies were each used to train a linear SVM with grid-tuned cost in [0.0001, 0.0002, …, 0.1000], in similar tenfold cross-validation. All other parameters were left to their defaults.

The trained models were then tested and evaluated on the testing set. Missing values in the testing set were interpolated as the mean methylation value of that CpG in the training set.

A two-sided test for Pearson’s correlation coefficient on the logit predicted probability of having cancer versus the log number of days in storage or number of days since last treatment plus one was used to check for the possible association of storage duration or treatment effects and model performance, respectively (Additional file 1: Figure S4).

### Enrichment analysis

Enrichment analysis of transcription factors that bind to the genomic loci of selected CpGs was analyzed using the UniBind Enrichment Analysis webtool (https://unibind.uio.no/enrichment/, accessed 19 June 2023) [24]. The background set of genomic loci was configured to be the set of CpGs after removal of storage-correlated and treatment-correlated features. Aggregated *p*-values were computed using unweighted Lancaster *p*-value aggregation from the aggregation *R* package [25], and thereafter, *q*-values for each transcription factor were obtained by the qvalue *R/Bioconductor* package [26] applied on the Lancaster-aggregated *p*-values.

Enrichment analysis of pathways for genes associated with select CpGs was analyzed using the GOmeth function of the missMethyl *R/Bioconductor* package [27]. The background set of CpGs was also configured to be the set of CpGs after removal of storage-correlated and treatment-correlated features.

### Linear regression

For each cell type, a linear regression model was built to model that cell type’s estimated proportion as a function of CpG methylation from the selected CpGs. Using *R*, the combination of CpGs used in each linear model was chosen by bidirectional stepwise optimization of the model’s Bayesian information criterion, and *p*-values were adjusted for multiple testing by Benjamini–Hochberg correction.

## Results

### A methylation profile distinguishes breast cancer patients from non-cancer controls

Peripheral blood of 256 ethnic Chinese cancer patients recruited from genetic testing clinics and 268 age- and ethnicity-matched non-cancer controls (*n* = 524) was profiled for DNA methylation at 866,238 CpGs (Table 1). Pre-processing removed 181,575 sites known SNPs and then 29,823 sites with cross-reactive probes, resulting in 654,840 remaining high-quality CpGs. Six outlier patients were removed due to outlying values in principle component analysis and estimated cell-type composition (Additional file 1: Figures S1 and S2).

Feature selection was performed on a training set comprising 175 cancer patients and 187 non-cancer controls (*n* = 362) on BMIQ-normalized M-values, beginning with 649,688 CpGs with non-missing values in all training set patients. Thereafter, storage-correlated and treatment-correlated CpGs were removed, and the top 30,000 CpGs correlated with cancer were selected. Next, boruta feature selection chose 79 CpGs which were further finalized to a methylation profile comprising 51 CpGs via tenfold cross-validated random forest-simulated annealing (Additional file 2: Table S1). This final methylation profile of 51 CpGs was used to train an xgbTree machine learning algorithm with AUC of 0.902 in the training set (Fig. 1).

**Fig. 2:**
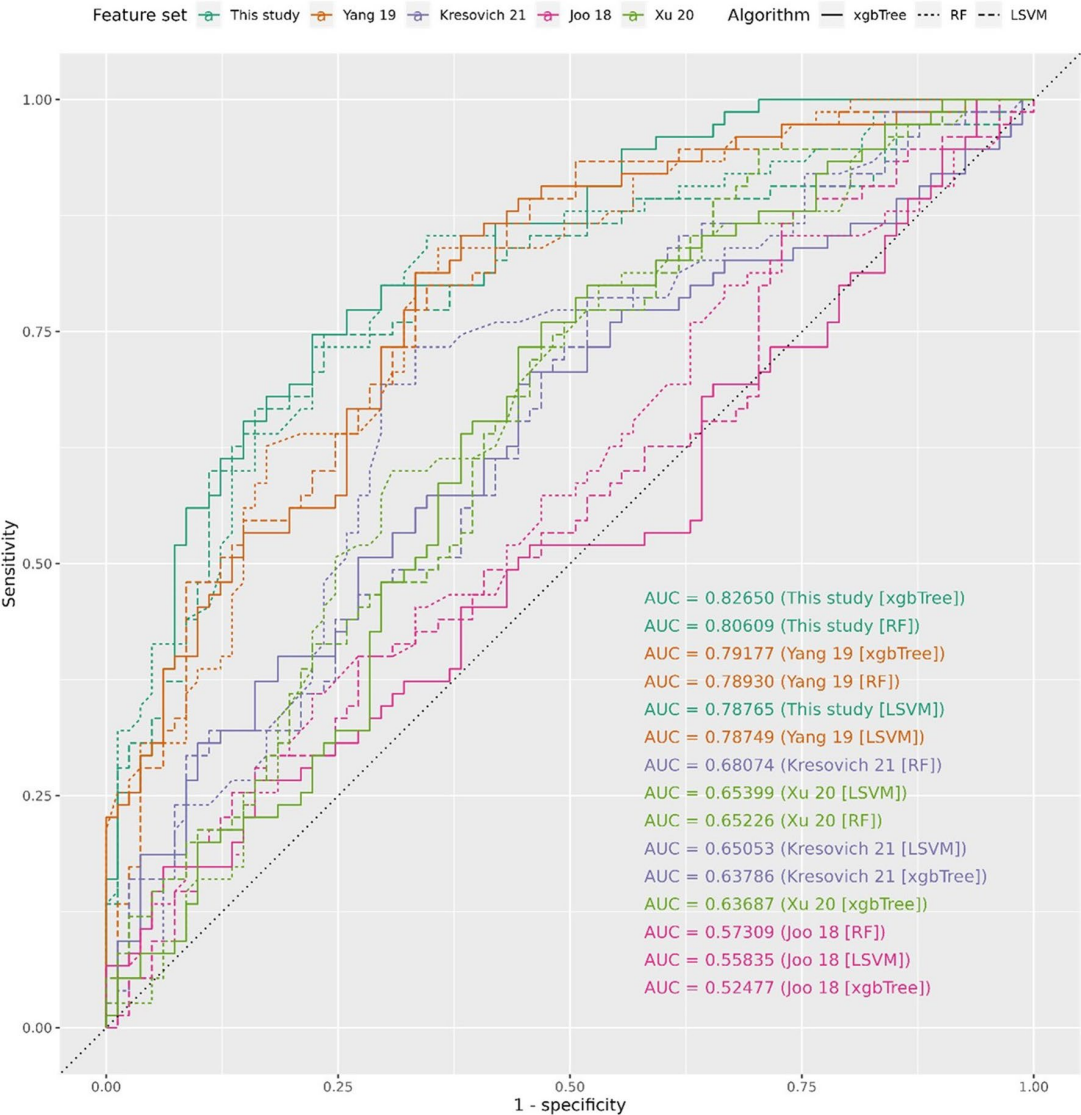
Predictive performance of each methylation profile paired with each algorithm when tested in an independent testing set, for the methylation profile from this study and profiles from four previous studies

**Fig. 3:**
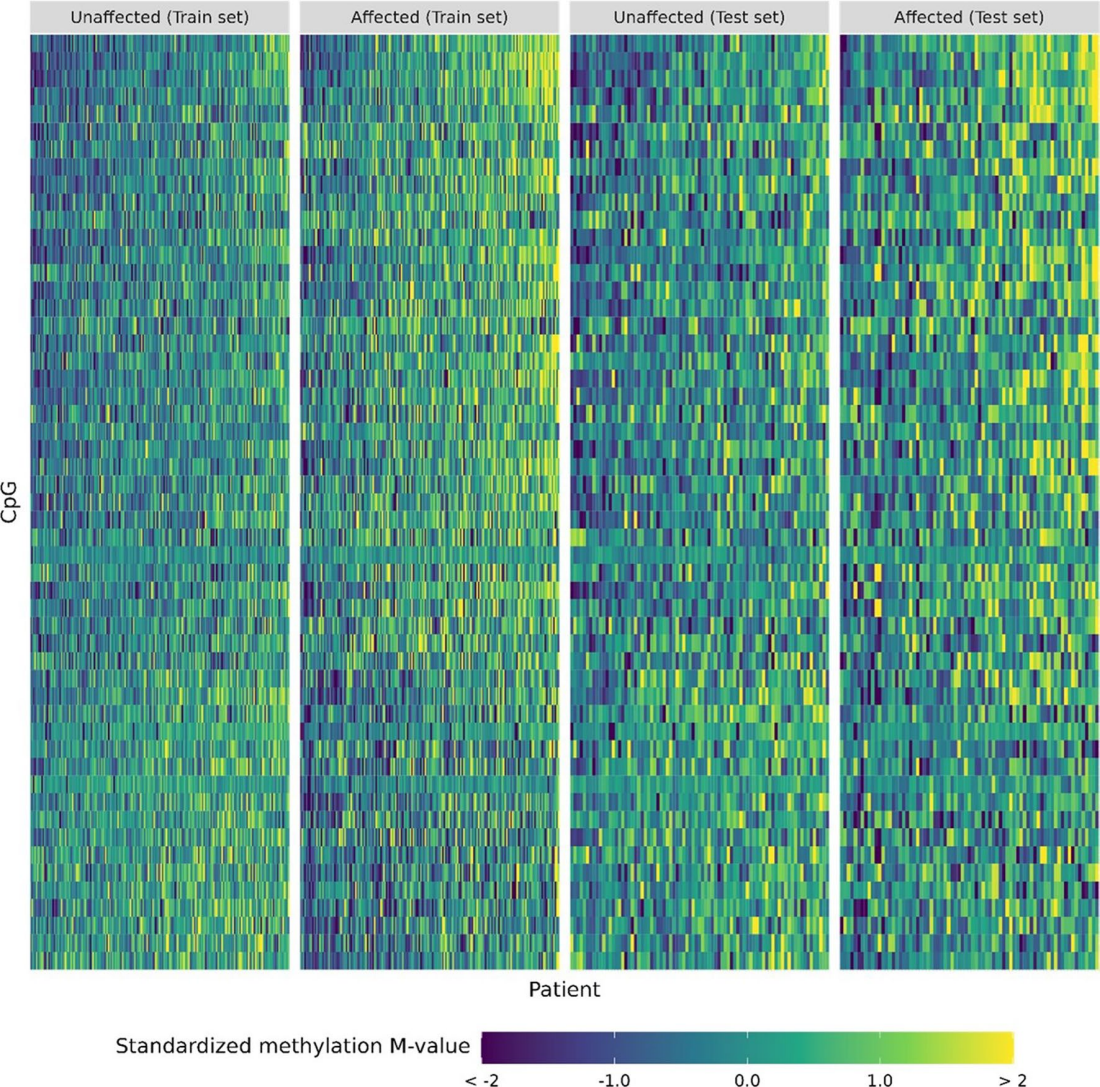
Heatmap of 51 CpGs in the selected methylation profile for unaffected and affected patients of the training set and of the testing set

On an independent testing set of 75 cancer patients and 81 non-cancer controls (*n* = 156), the selected methylation profile with xgbTree could distinguish cancer patients from healthy controls with 75% sensitivity and 78% specificity (AUC = 0.827, Figs. 2 and 3). We did not observe any evidence of confounding by treatment for cancer patients nor by the duration for which DNA samples were stored prior to methylation profiling (*p* ≥ 0.064, Additional file 1: Figure S4). The same methylation profile trained with a random forest (RF) machine learning algorithm did not perform as well as the xgbTree, nor did a methylation profile derived from linear SVM-simulated annealing paired with a linear SVM (LSVM) algorithm (Fig. 2).

### Better performance relative to previously identified breast cancer methylation profiles

The selected methylation profile of 51 CpGs outperformed four other sets of breast cancer methylation profiles identified previously in predominantly European populations (Fig. 2, Table 2, Additional file 1: Figure S5) [7–10], when trained on our training set and tested on our testing set of patients. The four previously identified methylation profiles were highly heterogeneous in performance, with the worst performing methylation profile performing only slightly better than by random chance (AUC = 0.525), and best performing methylation profile achieving a performance close to the best performing profile in this study (best AUC in previous methylation profiles = 0.792, best AUC in this study’s methylation profiles = 0.827) (Fig. 2).

**Table 2:** Four previous studies of breast cancer-associated blood methylation used for benchmarking

First author (year)	Features (orig. − unavail. here)	Cohort size (cases + controls)	Cohort country of origin	Pre-dx	Pre-tx
Yang 2019	450 − 26 CpGs	124,572 + 106,857	USA	Yes	Yes
Kresovich 2021	19 − 0 CpGs, 5 DNAm est	1090 + 851	United States, Italy	Yes	Yes
Xu 2020	72 − 4 CpGs	1371 + 1401	United States, Italy	Yes	Yes
Joo 2018	24 − 3 CpGs	87 + 123	Australia	Mixed	Unknown

orig., the original number of CpGs reported in that study; unavail., the number of CpGs in the originally reported list of CpGs which were not measured in this study due to differences in microarrays used; Pre-dx, blood was drawn before cancer diagnosis; Pre-tx, blood was drawn before cancer treatment; DNAm est., DNA methylation estimators from the DNA Methylation Age Calculator, each representing a single estimated quantity computed from many individual CpGs, such as DNA methylation age acceleration

Despite all four previous methylation profiles using the same HumanMethylation450 array, and the extensive overlap of HumanMethylation450 CpGs and the MethylationEPIC array used in this study, the specific CpG sites within the methylation profiles never overlapped with each other, except in expected cases where they were derived from similar feature selection steps by the same research group (Additional file 1: Figure S3). Predictive performance was stratified predominantly by methylation profile rather than the predictive model being used (Fig. 2). Limiting the testing set to only patients with no recorded history of any treatment, the selected methylation profile still outperforms the others (Additional file 1: Figure S6).

### Enrichment of immune-related transcription factors and pathways

In order to gain biological insight into the selected methylation profile of 51 CpGs, we tested for the enrichment of transcription factors binding to the genomic loci of those 51 CpGs by performing meta-analysis across the multiple transcription factor–DNA binding datasets for the human cell lines of the UniBind database [24]. After controlling for false discoveries, we identified three enriched transcription factors all from the AP-1 transcription family which function as regulators of the immune system: JUND, BATF3, and FOS [28]. Likewise, enrichment analysis of the functional pathways represented by genes in close proximity with the selected methylation profile of 51 CpGs identified an enrichment of immune-related pathways related to IL-12, IL-21, T_h_17 cell lineage commitment, and NK cell activation in the list of top ten enriched pathways (Table 3). There was no difference in the DNA methylation age acceleration of cancer patients and non-cancer controls (Additional file 1: Figure S7).

**Table 3:** Enrichment analysis for the selected methylation profile of 51 CpGs

Transcription factor enrichment analysis (showing aggregated *q*-value < 0.05)			
Transcription factor	Negative log10 *p*-values	Aggregated *p*-value	Aggregated *q*-value
*JUND*		1.46e-05	0.00391
*BATF3*		2.41e-04	0.03235
*FOS*		4.14e-04	0.03700
			

Pathway enrichment analysis (showing top 10 enriched pathways)		
Pathway	*p*-values	Genes in 51 CpGs (number of genes in pathway)
Positive regulation of interleukin-12 production	0.00057	*IL23A, MAPK11* (41)
Interleukin-21-mediated signaling pathway	0.00096	*IL21R* (1)
Response to interleukin-21	0.00096	*IL21R* (1)
Cellular response to interleukin-21	0.00096	*IL21R* (1)
Interleukin-12 production	0.00135	*IL23A, MAPK11* (61)
Regulation of interleukin-12 production	0.00135	*IL23A, MAPK11* (61)
Positive regulation of T helper 17 cell lineage commitment	0.00162	*IL23A* (4)
Regulation of defense response to virus	0.00261	*IL23A, CCDC92* (73)
Natural killer cell activation	0.00263	*IL21R, IL23A* (74)
Protein initiator methionine removal involved in protein maturation	0.00271	*METAP2* (2)

## Discussion

In this study, we have identified a breast cancer-associated methylation profile comprising 51 CpGs from Asian patients. In a machine learning algorithm, this methylation profile can distinguish Asian breast cancer cases from healthy controls better than previously reported breast cancer-associated methylation profiles. Enrichment analyses of transcription factor–DNA binding and functional pathways of genes associated with the 51 CpGs both suggest that the host immune response against cancer may play a role in driving the difference between methylation profiles of breast cancer cases and healthy controls.

Whereas the search for breast cancer-associated methylation profiles in peripheral blood DNA has mostly been targeted at cancer genes [6, 29, 30], our results suggest that the inclusion of DNA methylation of immune-related genes or pathways could improve the performance of peripheral blood screening for breast cancer. Our results saw an enrichment of IL-12, IL-21, T_h_17 cell lineage commitment, and NK cell activation pathways: Natural killer cells recognize and cytolyze tumor cells during normal immunosurveillance or as part of the immune response to tumors [31]. The potency of these NK cells is enhanced by both IL-12 and IL-21 [32, 33]. T_h_17 cells are a rare subset of T helper cells whose role in the tumor microenvironment is context-dependent and not yet well understood [34], though they have been observed to secrete IL-21 [35]. Conversely, IL-12 stimulates the expansion of T_h_17 cells [36]. Concurrently, there was an enrichment of transcription factors from the multifunctional AP-1 transcription factor family, which has roles in different aspects of the immune system including in the immune response against cancer [28]. In mice, knockout of BATF3 results in more metastases in a NK cell-dependent manner [37] and regulates the activity of T_h_17 through IL-12 production though this regulation was not demonstrated in the context of cancer [38]. Furthermore, we found that the estimated cell-type proportions of the samples could be predicted using the 51 CpGs of the selected methylation profile (Additional file 2: Table S2). All these suggest that the DNA methylation profile identified in this study reflects the host immune response against cancer, to such an extent that it can distinguish breast cancer cases from healthy controls.

The selected methylation profile identified here, with an AUC of 0.823, outperforms four others previously identified in the literature [7–10]. We note that the improvement in performance is modest when compared to the best previous study (AUC = 0.792) [9], despite this study utilizing almost twice as many CpGs on the MethylationEPIC array. This could suggest that further improvements in CpGs coverage are not as important as developing better algorithms or incorporating additional modalities of data. Furthermore, the best performing previously reported breast cancer-associated methylation profile was trained on a predominantly European population [9] yet performed reasonably well in our cohort of Asian patients. This suggests that methylation biomarkers can be generalized from one population to another within reason, though further study is required to characterize the extent of loss of performance. 

The lack of replication among CpG sites identified in previous studies as well as in our own may stem from various factors, including methodological disparities in sample processing, data analysis, and statistical approaches, as well as differences in study designs and sample characteristics, such as population demographics. Heterogeneity in genetic backgrounds or environmental exposures could also contribute to these discrepancies. Given the low reproducibility observed in epigenome-wide association studies (EWAS), it is essential to perform single-assay validation, such as pyrosequencing, quantitative methylation-specific PCR, and other complementary techniques, to independently confirm the findings of EWAS. These validation efforts serve to enhance the robustness and reliability of the identified CpG sites and their associations with breast cancer.

Our study is limited due to possible confounding from storage duration or treatment effects. However, we have tried to alleviate these effects in feature selection by removing storage-correlated and treatment-correlated CpGs. Indeed, we have shown that the predictive model is not associated with any of these confounders. It should be noted as well that a minority of CpGs of previously identified breast cancer-associated methylation profiles were not included in the benchmark as they are not covered in the MethylationEPIC array (Table 2). Additionally, using an orthogonal method to verify the immune-related DNA methylation profile would have been desirable, but this was not possible due to insufficient blood samples. To establish the specificity of these markers in a screening population, it will be crucial to assess the identified methylation profile in larger breast cohorts and in patients with other cancer types. Finally, the patients in our cohort were recruited from various genetic testing clinics such that all had either early-onset and/or a family history of breast cancer, so the applicability of these results to early detection of sporadic breast cancer should be taken with care.

## Conclusions

The development of an accurate blood-based biomarker assay for early detection of breast cancer has the potential to drastically reduce the costs associated with false positives and overdiagnosis in current screening programs, and more importantly overall breast cancer mortality. To that effect, we have identified a whole blood methylation profile with better predictive performance in benchmark against previously identified methylation profiles. Furthermore, we provide evidence for a plausible mechanism in the immune response against cancer as the driver behind the association of whole blood-methylation profiles and breast cancer.

### Supplementary Information

**Additional file 1**: **Fig. S1.** PCA of M-values identifies outliers. **Fig. S2.** Predicted cell-type composition identifies outliers. **Fig. S3.** Overlap of CpGs from previous studies and from our study. The feature selection of Kresovich 2019 starts with features from Xu 2020, explaining their overlap. **Fig. S4.** Performance of the predictive model on the independent testing set is not correlated with storage duration nor with time since last treatment, at p ≥0.064. **Fig. S5.** Heatmap of methylation profiles from (A) Joo et al. 2018, (B) Xu et al. 2020, (C) Yang et al. 2019, and (D) Kresovich et al. 2021. The “DNAm estimators” used in Kresovich et al. 2021 are not shown here. **Fig. S6.** Predictive performance across multiple methylation profiles and algorithms in the independent testing set, including profiles from this study and four previous studies, for patients without a recorded history of any treatment. **Fig. S7.** Estimated DNA methylation age versus chronological age across affected patients and unaffected controls. The systematic bias in the estimated DNA methylation age as compared to chronological age may be explained by the difference between HumanMethylation450, the microarray used to train the DNA methylation age estimator, and MethylationEPIC, the microarray used in our study.

**Additional file** **2**: **Table S1.** 51 CpGs of the selected methylation profile identified using the MethylationEPIC array. **Table S2.** Linear regression models using CpGs from the 51 CpGs of the selected methylation profile can predict cell-type composition.

## Data Availability

De-identified data from the in-house cohort of breast cancer patients and healthy controls profiled in this study are available at NCBI’s Gene Expression Omnibus (GEO) with accession number GSE243529 (https://www.ncbi.nlm.nih.gov/geo/query/acc.cgi?acc=GSE243529).

## Abbreviations

AUC: Area under the curve
DNAm: DNA methylation
DNAmAge: DNA methylation age
NK: Natural killer
PCA: Principal component analysis
RF: Random forest
ROC: Receiver operating characteristics
SVM: Support vector machine
T_h_17: T helper 17 cells
