# Supplementary material for: Machine learning unveils an immune-related DNA methylation profile in germline DNA from breast cancer patients
Source: Clin Epigenetics. 2024 May 15;16:66. doi: 10.1186/s13148-024-01674-2 (PMC11094860; doi:10.1186/s13148-024-01674-2)

**Additional file 1: Supplementary Figures**

**Supplementary Figure S1.** PCA of M-values identifies outliers.

**
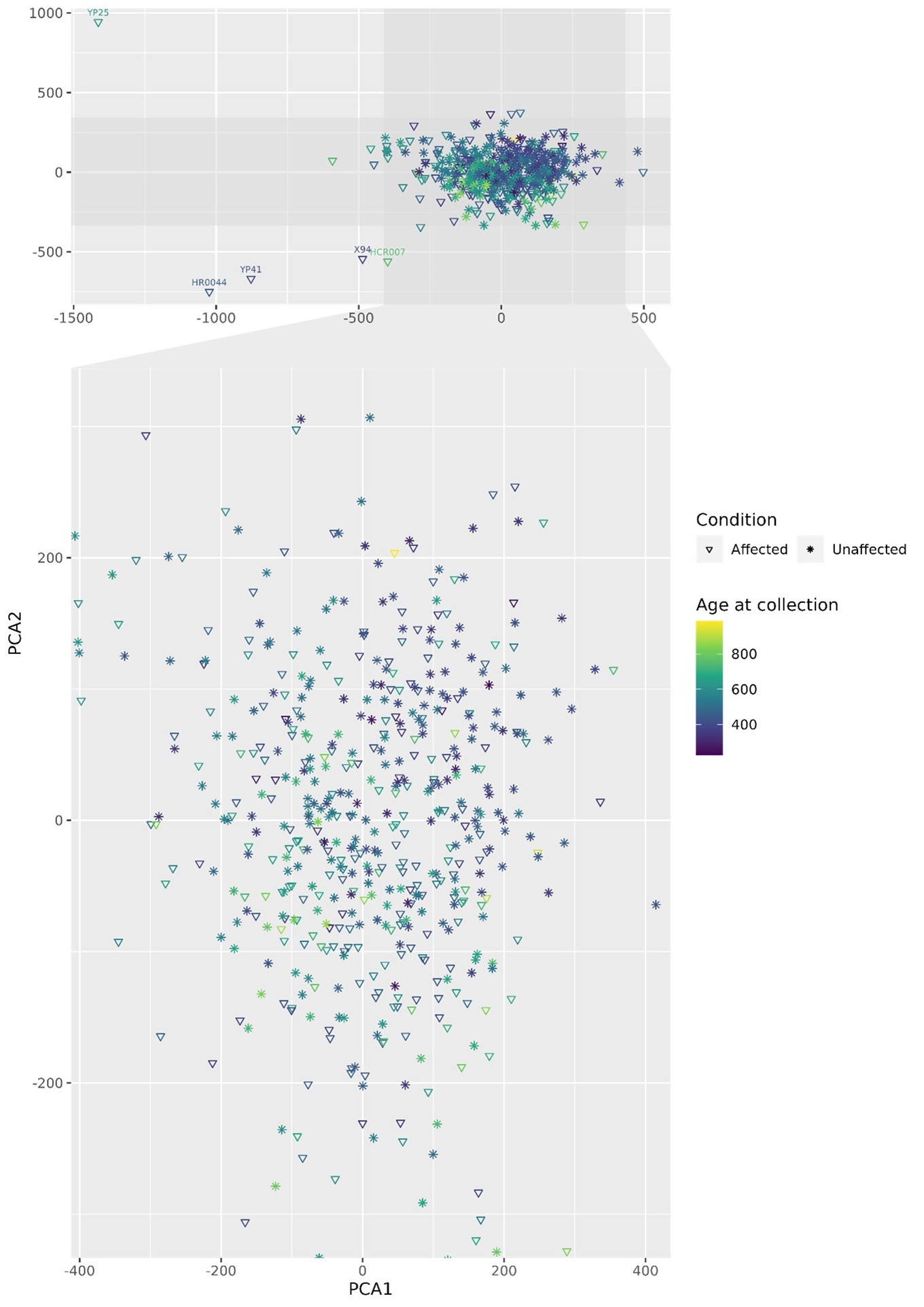
**

**Supplementary Figure S2.** Predicted cell-type composition identifies outliers.

**
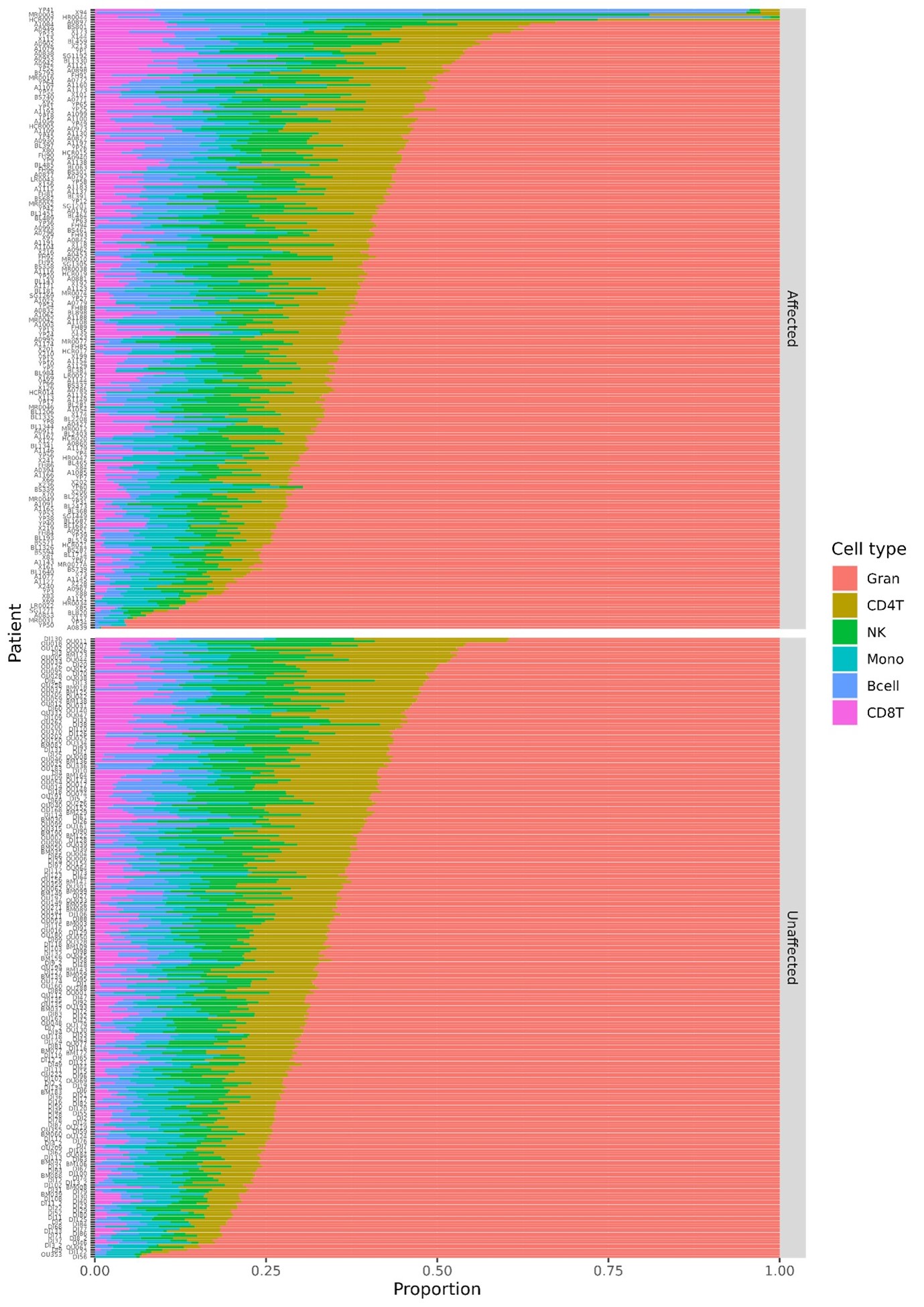
**

**Supplementary Figure S3.** Overlap of CpGs from previous studies and from our study. The feature selection of Kresovich 2019 starts with features from Xu 2020, explaining their overlap.


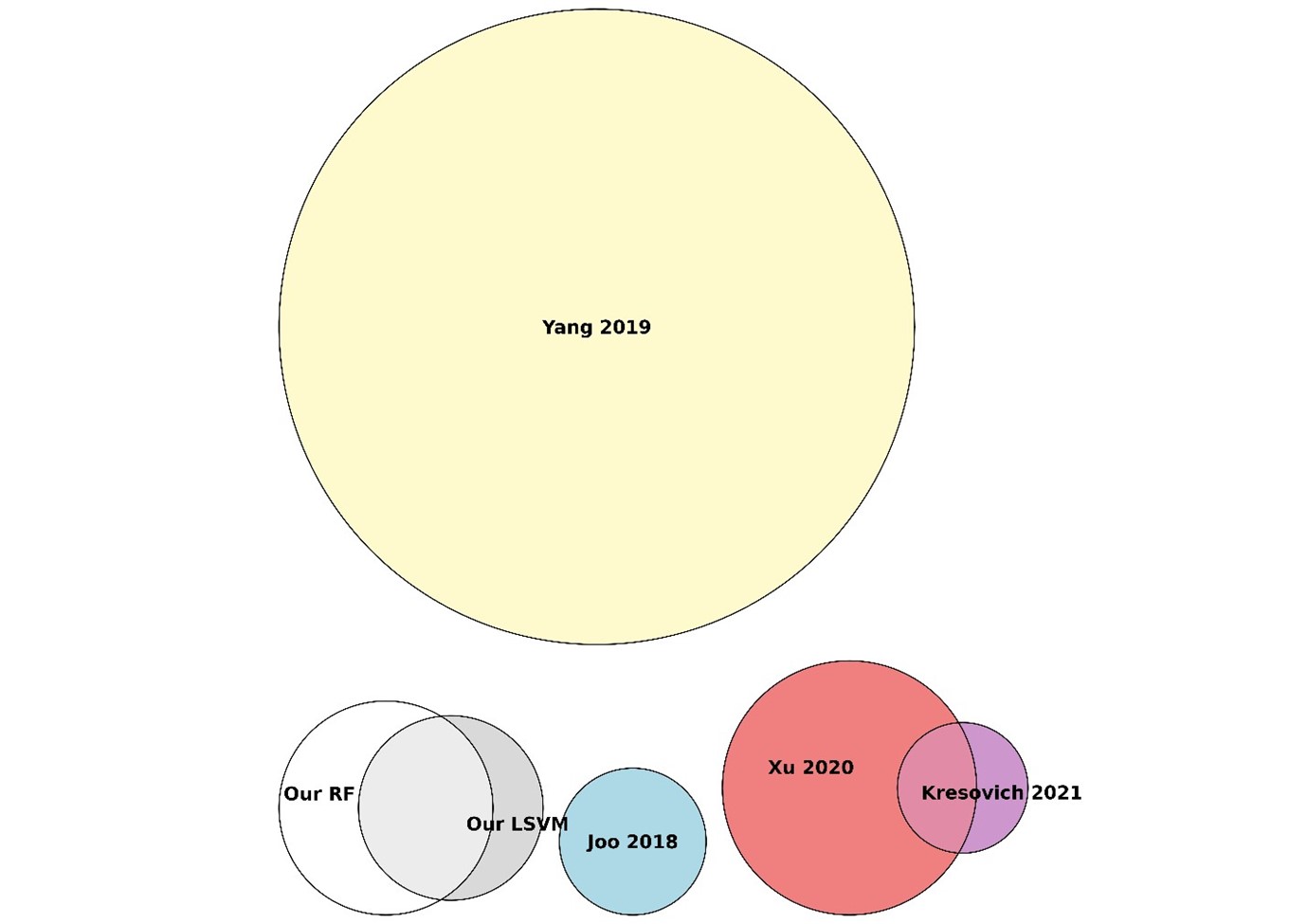


**Supplementary Figure S4.** Performance of the predictive model on the independent testing set is not correlated with storage duration nor with time since last treatment, at p ≥0.064.


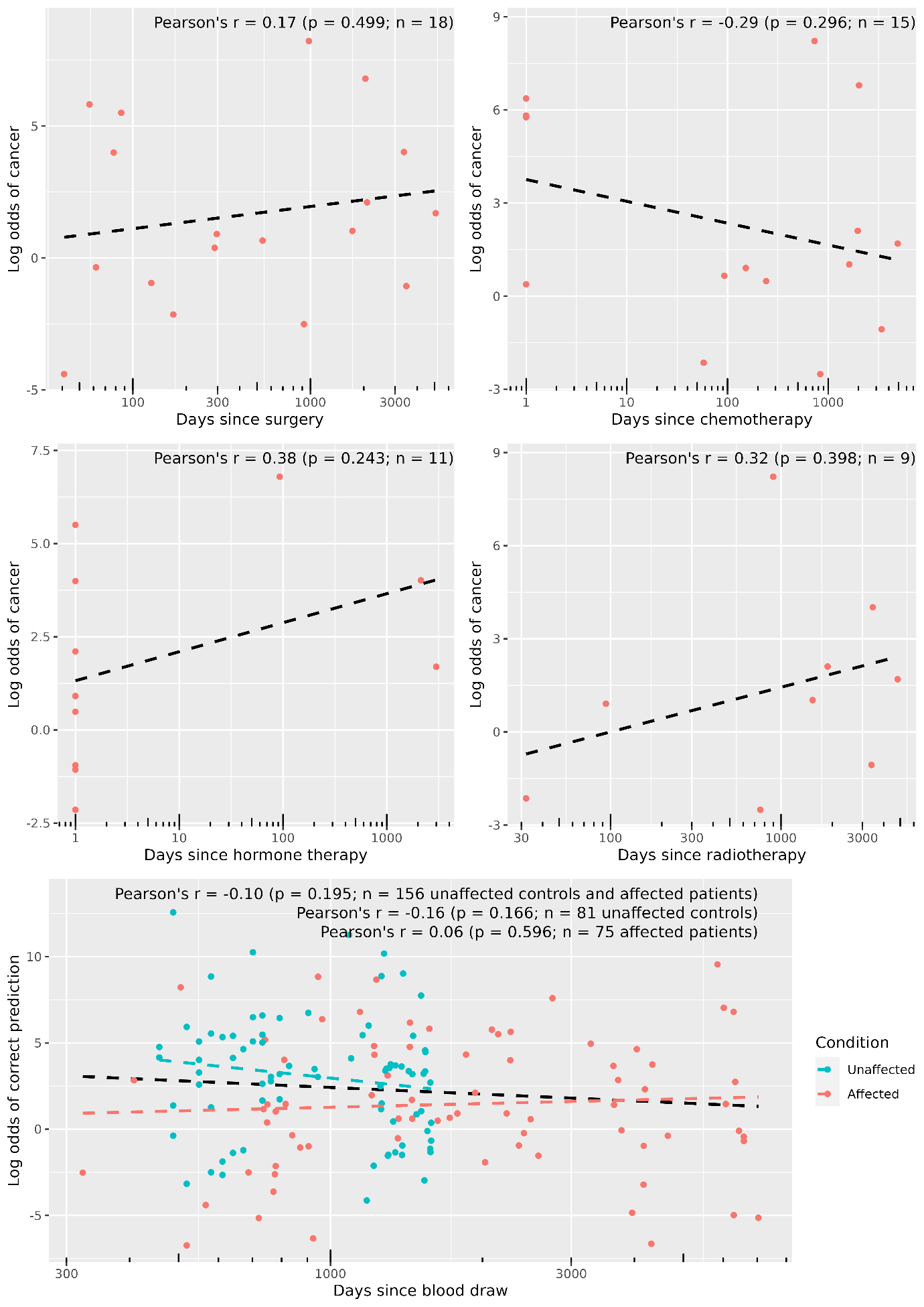


**Supplementary Figure S5.** Heatmap of methylation profiles from (A) Joo et al. 2018, (B) Xu et al. 2020, (C) Yang et al. 2019, and (D) Kresovich et al. 2021. The “DNAm estimators” used in Kresovich et al. 2021 are not shown here.


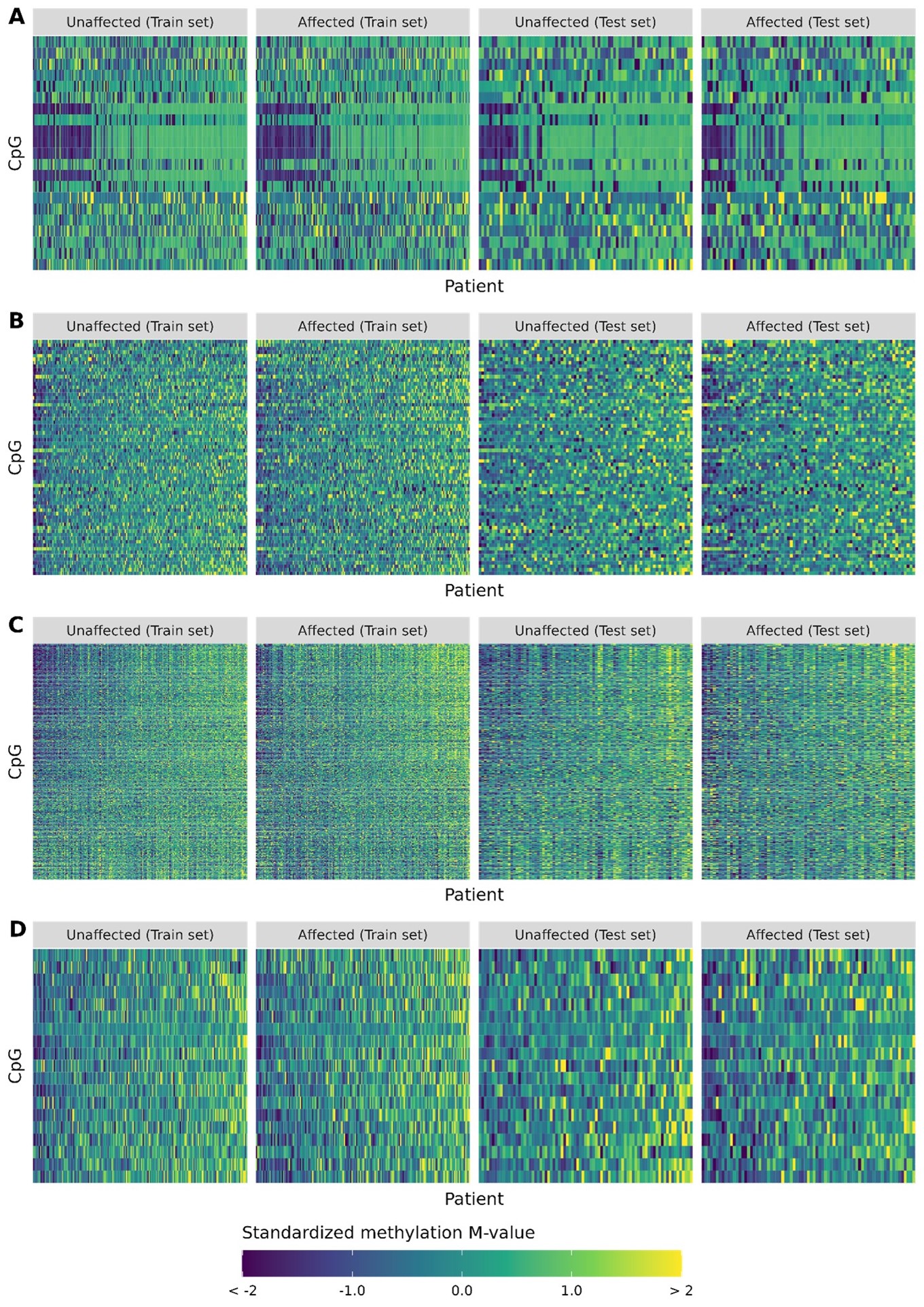


**Supplementary Figure S6.** Predictive performance across multiple methylation profiles and algorithms in the independent testing test, including profiles from this study and four previous studies, for patients without a recorded history of any treatment.

**
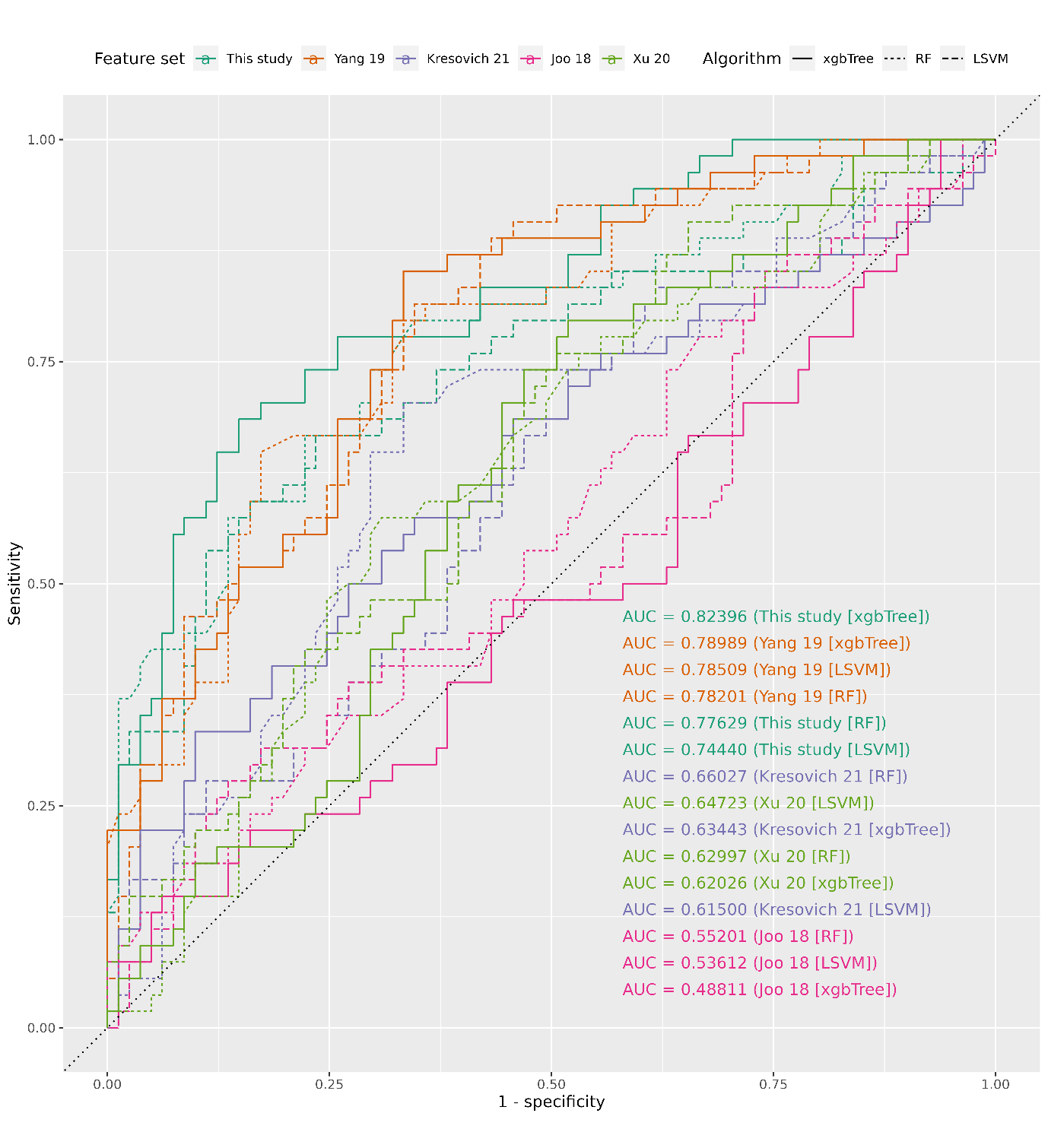
**

**Supplementary Figure S7.** Estimated DNA methylation age versus chronological age across affected patients and unaffected controls. The systematic bias in the estimated DNA methylation age as compared to chronological age may be explained by the difference between HumanMethylation450, the microarray used to train the DNA methylation age estimator, and MethylationEPIC, the microarray used in our study.


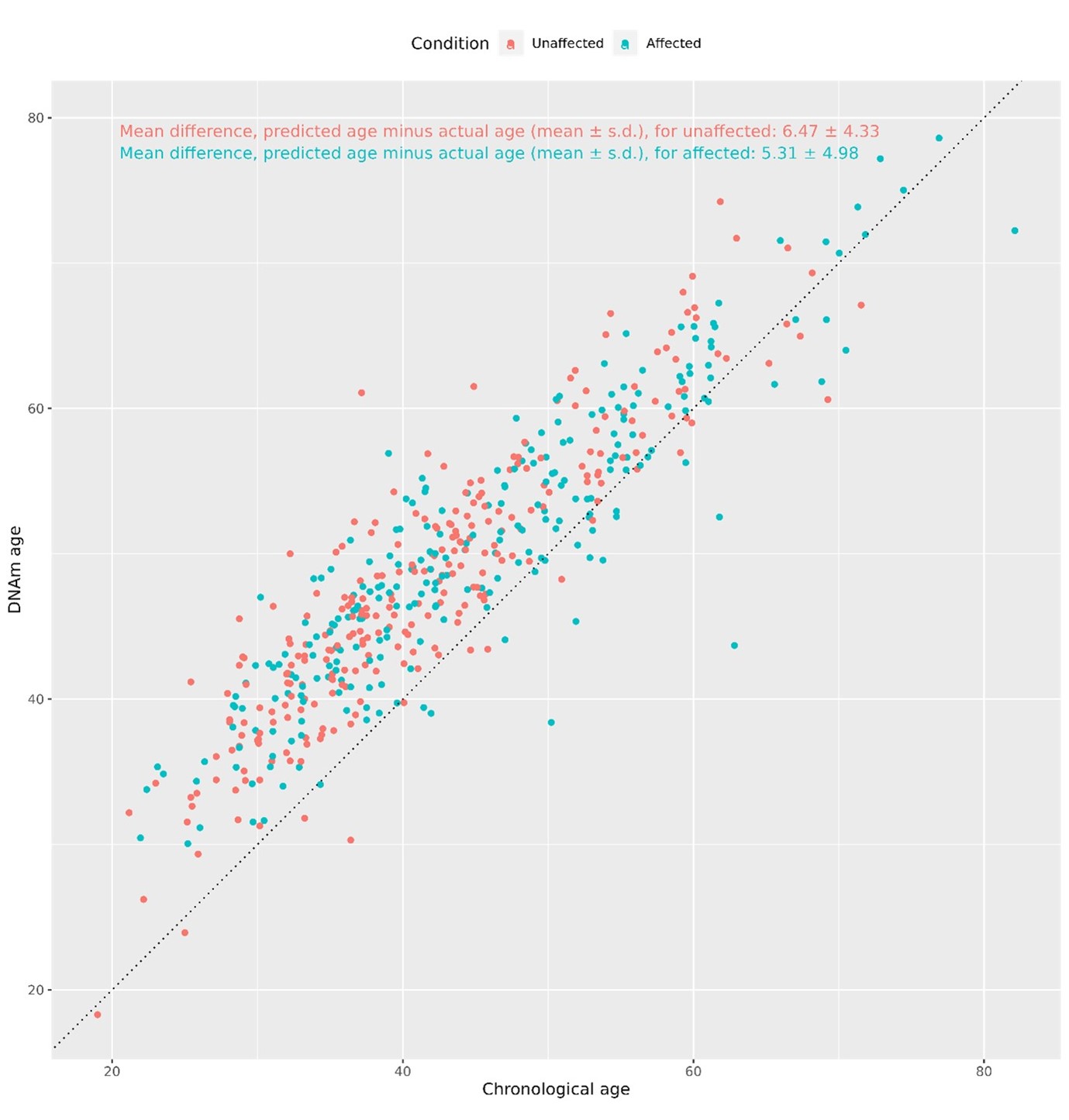

Supplement: Supplementary file 1 — Additional file 1: Fig. S1. PCA of M-values identifies outliers. Fig. S2. Predicted cell-type composition identifies outliers. Fig. S3. Overlap of CpGs from previous studies and from our study. The feature selection of Kresovich 2019 starts with features from Xu 2020, explaining their overlap. Fig. S4. Performance of the predictive model on the independent testing set is not correlated with storage duration nor with time since last treatment, at p ≥0.064. Fig. S5. Heatmap of methylation profiles from (A) Joo et al. 2018, (B) Xu et al. 2020, (C) Yang et al. 2019, and (D) Kresovich et al. 2021. The “DNAm estimators” used in Kresovich et al. 2021 are not shown here. Fig. S6. Predictive performance across multiple methylation profiles and algorithms in the independent testing set, including profiles from this study and four previous studies, for patients without a recorded history of any treatment. Fig. S7. Estimated DNA methylation age versus chronological age across affected patients and unaffected controls. The systematic bias in the estimated DNA methylation age as compared to chronological age may be explained by the difference between HumanMethylation450, the microarray used to train the DNA methylation age estimator, and MethylationEPIC, the microarray used in our study. [file 13148_2024_1674_MOESM1_ESM.docx]
